# Cardiovascular Aging and Physical Activity: Insights From Metabolomics

**DOI:** 10.3389/fcvm.2021.728228

**Published:** 2021-09-20

**Authors:** Ryan Mao Heng Lim, Angela S. Koh

**Affiliations:** ^1^National Heart Centre Singapore, Singapore, Singapore; ^2^Duke-National University of Singapore Medical School, Singapore, Singapore

**Keywords:** aging, cardiovascular disease, metabolomics, physical activity, cardiovascular trial, musculoskeletal function

## Abstract

The purpose of this review is to explore how metabolomics can help uncover mechanisms through which physical activity may influence the progression of cardiovascular aging. Cardiovascular aging is a process of functional and structural changes in older adults which can progress to cardiovascular disease. Metabolomics profiling is an investigative tool that can track the diverse changes which occur in human biochemistry with physical activity and aging. This mini review will summarize published investigations in metabolomics and physical activity, with a specific focus on the metabolic pathways that connect physical activity with cardiovascular aging.

## Introduction

The role of physical activity in reducing the risks of cardiovascular disease (CVD) is well-established (1–6). Physical activity has an impact on many of the traditional cardiovascular risk factors including high blood pressure, type 2 diabetes mellitus and abnormal lipid profiles (7–12). Physical activity has been shown to be a major modifiable lifestyle factor that can mitigate aging related changes in cardiovascular function as well as sarcopenia and frailty (13). Physical activity thus has an important role in modulating highly prevalent diseases such as obesity, type 2 diabetes mellitus, cardiovascular disease as well as aging-related declines in muscle mass and overall function. In recognition of the important beneficial effects of physical activity, healthcare practitioners are now advocating exercise as a medicine. Given the potential importance of physical activity as a therapeutic tool, we need to gain a better understanding of the underlying biological and physiological processes stimulated by physical activity in order to elucidate how it works as an intervention. Uncovering the mechanisms for the beneficial effects of physical activity will improve our basic understanding of disease pathophysiology, highlight new potential pathways for intervention and identify biomarkers to help guide exercise prescriptions. Current evidence points to the importance of fuel metabolism and mitochondrial oxidation pathways for physical activity effects on cardiovascular health (14, 15). Metabolomics, defined as the study of chemical processes involving metabolites within the human biological system, can serve as a useful tool to guide further investigative work in these areas.

## Metabolic Alterations in Human Serum With Physical Activity

Physical activity increases energy demand across multiple tissues and stimulates acute and chronic changes in metabolic pathways. These changes can be detected through metabolomics analysis of serum. In the first 24 h after a bout of exercise lactate, pyruvate, TCA cycle intermediates, fatty acids, acylcarnitines, and ketone bodies all typically increase whereas bile acids decrease (16). Multiple trials have shown that chronic exercise also leads differences in metabolomic profiles. Exercise groups generally have lower levels of triglycerides, higher levels of high-density lipoprotein cholesterol and apolipoprotein A1 and decreased insulin resistance, fasting insulin levels and glycosylated hemoglobin A1c as compared to control groups. Exercise groups also have higher levels of interleukin-18 and lower levels of leptin, fibrinogen, and angiotensin II (17). Additionally, glycerol concentration is directly correlated and glutamine is indirectly correlated with resting heart rate (18) which is known to be an independent predictor of exercise capacity (19) and cardiovascular outcomes (20). Pang et al. conducted a comprehensive case-control study that aimed to determine the effect of physical activity on circulating metabolomics and subsequent incidence of cardiovascular disease. Increased physical activity was inversely correlated with biomarkers such as VLDL, LDL, alanine, lactate, acetoacetate, and the inflammatory marker glycoprotein acetyls, which were in turn highly associated with cardiovascular disease (21). A common finding of these studies is the prominent response of the major fuel metabolic pathways (fatty acids, pyruvate/lactate, alanine, glutamine) as well as central carbon and mitochondrial metabolism (TCA cycle intermediates, acylcarnitines, and acetoacetate) to exercise interventions.

## Cardiac Response to Physical Activity

The increased energy demand from physical activity alters metabolic pathways across multiple tissues. The most notable changes occur in mitochondria, fuel oxidation and muscle function pathways. The typical cardiac response to physical activity includes physiologic growth and alteration of metabolic pathways (22). Fatty acid oxidation, electron transport, and TCA cycle genes are all up regulated by physical activity. These changes are distinct from the metabolic changes associated with heart failure which include pathophysiologic remodeling, reduced fatty acid and mitochondrial fuel oxidation and increased reliance on glucose (23).

Treating human myotubes with natriuretic peptide treatment increased oxidative phosphorylation genes, recapitulating the effect of exercise training on muscle fat oxidative capacity *in vivo*. This was accompanied by positive correlations between natriuretic peptide receptor type A gene expression and mRNA levels of PPAR coactivator-1 (PGC1a) and oxidative phosphorylation genes in human skeletal muscle (24). In an interventional human study, 12 weeks of twice daily supplementations of a beverage containing L-carnitine and carbohydrate were given to men after low intensity physical activity. L-carnitine is a quaternary amine which facilitates mitochondrial fatty acid burning. Compared to controls, study subjects who received supplementation had upregulation of genes related to fuel metabolism after 12 weeks. These genes represented pathways of insulin signaling, peroxisome proliferator-activated receptor signaling and fatty acid metabolism (25).

## Cardiovascular Health and Metabolism

Many of the metabolites altered by physical activity have also been linked to cardiovascular disease risk. These metabolites are generated by pathways which may provide the mechanistic link between physical activity and reduction in cardiovascular risk. Observational studies have noted that serum free fatty acids, the acylcarnitines, and amino acid levels are associated with cardiovascular health markers (26). In older subjects, medium and long-chain dicarboxyl and hydroxyl acylcarnitines levels were directly associated with higher arterial stiffness (27), while greater accumulation of wide-spectrum acyl-carnitines, alanine and glutamine/glutamate were associated with lower aerobic capacity as measured by VO2, which is the maximum rate of oxygen consumption measured during exercise of increasing intensity (28). However, ethnicity and genetics may also have a role to play in metabolic profile and cardiovascular health. In an article by Benedetti et al., South Asian men had substantially lower levels of cardiovascular fitness as compared to white European men even though there were no significant differences in physical activity or sedentary behavior. The authors found that South Asian men exhibited higher concentrations of five free fatty acids (FFA), elevated fasting insulin, interleukin-6, and lower fasting HDL-C among other differences (29). Despite having similar levels of physical activity, differences among ethnic groups in cardiovascular health may be explained by intrinsic differences in metabolic profiles/pathways. The effect of some of the highlighted metabolites on cardiovascular health can be linked to well-described mechanisms such as VLDL and LDL accumulation predisposing to atherosclerosis. Many of the other metabolites associated with cardiovascular risk (free fatty acids, amino acids, and acylcarnitines) are intermediates in fuel metabolism and mitochondrial oxidation pathways. It is still unclear how these pathways are linked to cardiovascular risk. However, since these pathways can be modified by physical activity they may serve as a common link that explains how physical activity might improve cardiovascular health.

## Tricarboxylic Acid (TCA) Cycle

The TCA cycle is known to be upregulated when there is a high demand for ATP. Increased energy demand stimulates regulatory enzymes of the cycle such as isocitrate dehydrogenase and alpha-ketoglutarate dehydrogenase. It has also been shown in recent studies that there is a rise in the level of TCA cycle intermediates just after an acute bout of physical activity (16). Certain amino acids such as alanine and glutamine/glutamate can serve as metabolic fuels by feeding into the TCA cycle. The process by which amino acids are fed into the TCA cycle is known as “anaplerosis” (or “filling of mitochondria”). Koh et al. have reported that low serum levels of anaplerotic amino acids are associated with better cardiorespiratory fitness (CRF; VO2) in human cohorts (28). The observed reduction in anaplerotic amino acid levels could be due to increased consumption by an activated TCA cycle. The importance of matching of carbon fuel inflow and TCA cycle activity has been replicated in a study that examined metabolic changes in the heart in response to heart failure or physical activity (30). In heart failure there was an elevation of lactate and acylcarnitines with a reduction in TCA cycle intermediates. In contrast, exercised hearts showed decreases in both acylcarnitines as well as TCA cycle intermediates. The former result suggests accumulation of carbon fuel which is not able to be cleared by a slowing TCA cycle. The latter suggests increased consumption of carbon fuel as a result of higher TCA cycle activity. These findings highlight a role for increased activity of the TCA cycle brought about by sustained aerobic training, thereby improving VO2 levels and links TCA cycle activity to cardiorespiratory fitness.

## Lipids

Free fatty acids (FFAs) and its associated metabolites are associated with insulin resistance, diabetes mellitus and coronary artery disease, mainly due to the accumulation of mitochondrially derived by-products of lipid oxidation in skeletal muscle. When muscle tissue is exposed to elevated lipids chronically, there is an increase rather than decrease in expression of genes of the fatty acid β-oxidative pathway. However, this causes a disconnect between mitochondrial beta-oxidation and TCA cycle activity, resulting in build-up of beta-oxidative metabolites and reactive oxygen species (ROS) that promote insulin resistance (31). Rodent models support the theory that physical activity improves mitochondrial and TCA cycle activity by increasing expression levels of peroxisome proliferator-activated receptor-gamma co-activator 1alpha (PGC1alpha), which enables muscle mitochondria to better cope with a high lipid load (32). This thereby reduces accumulation of metabolites from lipid oxidation, preventing the development of insulin resistance. Additionally, accumulation of lipid intermediates are directly responsible for cardio lipotoxicity and ventricular dysfunction, thereby contributing to poor cardiovascular health (33).

## Amino Acids

Separately, a previous study has also drawn an association between increased branched chain amino acids (BCAA) levels (specifically leucine and isoleucine) and prevalence of heart failure. Of note, diabetes mellitus was present in about 70% of heart failure patients in this study, which may act as an intermediate step to the development of heart failure (34). This finding is supported by other studies that demonstrate a clear association between BCAA and related metabolites with insulin resistance, perhaps even more so than the association between FFAs and insulin resistance (35, 36). Physical activity may decrease BCAA levels by increasing the uptake of BCAAs into the TCA cycle as anaplerotic substrates. This reduces the accumulation of BCAAs which is associated with heart failure.

Recently, Cheng et al. (37) also demonstrated that a panel of metabolites including histidine, phenylalanine, spermidine and phosphatidylcholine C34:4, has diagnostic value in heart failure similar to B-type natriuretic peptide (BNP). Another metabolite panel consisting of the asymmetric methylarginine/arginine ratio, butyrylcarnitine, spermidine, and the total amount of essential amino acids also significantly prognosticate for death or heart failure-related re-hospitalization.

## Metabolic Flexibility

The heart primarily derives its contractile energy from the oxidation of fatty acids (38). However, during acute haemodynamic stress the heart responds by switching to using glucose as a fuel (39). The ability to switch from fatty acid to carbohydrate-derived fuel sources is termed ‘metabolic flexibility'. Gibbs et al. showed that healthy, exercise-trained mice underwent a physiologic metabolic switching in the heart with decreased glycolytic activity. This reduced reliance on glucose was associated with increased physiological remodeling. In the failing heart, this metabolic flexibility is reduced and glycolysis predominates even in the absence of haemodynamic stress, with reduction in respiratory chain activity and impaired reserve for mitochondrial oxidative flux (40, 41). Other work in mice involved cardiac-specific expression of a kinase-deficient 6-phosphofructo-2-kinase/fructose-2,6-bisphosphatase transgene. These mice have constitutionally low glycolytic activity. The hearts from these mice had enhanced function and larger myocytes. This enforced metabolic change was also associated with modest mitochondrial damage that was not seen in the wild type mice. These studies demonstrate that the optimal fuel for hearts to use is context dependent. Maneuvers which limit the heart's ability to select the desired fuel are associated with defects in heart function. The overall conclusion from this work is that metabolic flexibility is important for maintaining both mitochondrial health and normal tissue function in the heart (42).

Exercise training may induce changes in the genomic and proteomic level to increase capacity for substrate utilization and metabolic flexibility in both cardiac and skeletal muscle (43). Physical activity may also improve the efficiency of fatty acid oxidation by means of upregulating the TCA cycle (as elucidated upon above) and enhancing oxidative metabolism. This reduces the reliance of anaerobic glycolysis to generate energy in the heart as more frequently observed in cardiomyopathic hearts. Physical activity decreases baseline lactate levels by promoting the efficiency of oxidative metabolism and metabolic flexibility. Blood lactate levels have been found to be associated with atherosclerosis (44). A decrease in baseline blood lactate levels may thereby reduce blood pressure, vascular dysfunction, and improve blood flow to the heart. This thereby increases the oxygen supply to the heart, allowing for increased oxidative metabolism and decline in glycolysis rates. Lactate is thus further reduced, and the positive cycle would bring about superior cardiovascular health.

The importance of metabolic flexibility for cardiovascular health suggests that dynamic testing of the metabolic response to physical activity and/or hemodynamic stress may be a novel research and clinical tool to assess cardiovascular fitness.

## Impact of Physical Activity on Cardiovascular Health in Aging and Aging Skeletal Muscle

For sedentary old adults that are frail or pre-frail, physical activity can improve cardiorespiratory fitness, muscle strength, functional status and quality of life (45, 46). After an adverse cardiovascular event such as myocardial infarction, physical activity reduces further decline in function and worsening frailty (47–49). Aerobic exercise training also confers benefit in aerobic capacity to healthy older adults (48) as well as older heart failure patients (50) with reduced ejection fraction (51) or preserved ejection fraction (52). Notably, a recent multicentre randomized control trial in older patients hospitalized for acute decompensated heart failure revealed that a tailored progressive rehabilitation intervention that included multiple physical function domains such as strength, balance, mobility and endurance resulted in a greater improvement in physical function than usual care. These greater improvement in physical function relative to the control group were seen despite the control group receiving routine physical or occupational therapy or traditional cardiac or pulmonary rehabilitation. This suggests that comprehensive physical activity including both aerobic and strengthening exercise may be a valuable tool in rehabilitation for older heart failure patients (53).

Mitochondrial function and energetics are central to the maintenance of good musculoskeletal mass and the prevention of aging-related muscle atrophy (54). In aging muscle, there is both a decline in mitochondrial organelle content and impaired mitochondrial function (55, 56). Some studies suggest that declines in mitochondrial respiratory function are associated with reduced levels of physical activity and are not directly tied to aging. Melov et al. showed that genes implicated in mitochondrial function and metabolism were among 306 genes found to be reduced with age. After 6 months of resistance training, these genes normalized toward a younger transcriptomic signature (57). Maintaining healthy muscle metabolism is thus important for prevention of age-related decline in muscle mass and function. Muscle metabolism is also important for prevention and treatment of type 2 diabetes and related metabolic disease. Skeletal muscle is especially important in the disposal of intravenous glucose by increasing glucose storage and use (58). Chronic exercise increases insulin sensitivity even in older humans by increasing skeletal muscle GLUT-4 abundance (59) and capillarisation (60). The beneficial effects of physical activity on skeletal muscle and whole body insulin sensitivity has been demonstrated in multiple animal (61) and human (62) trials.

**Figure 1:**
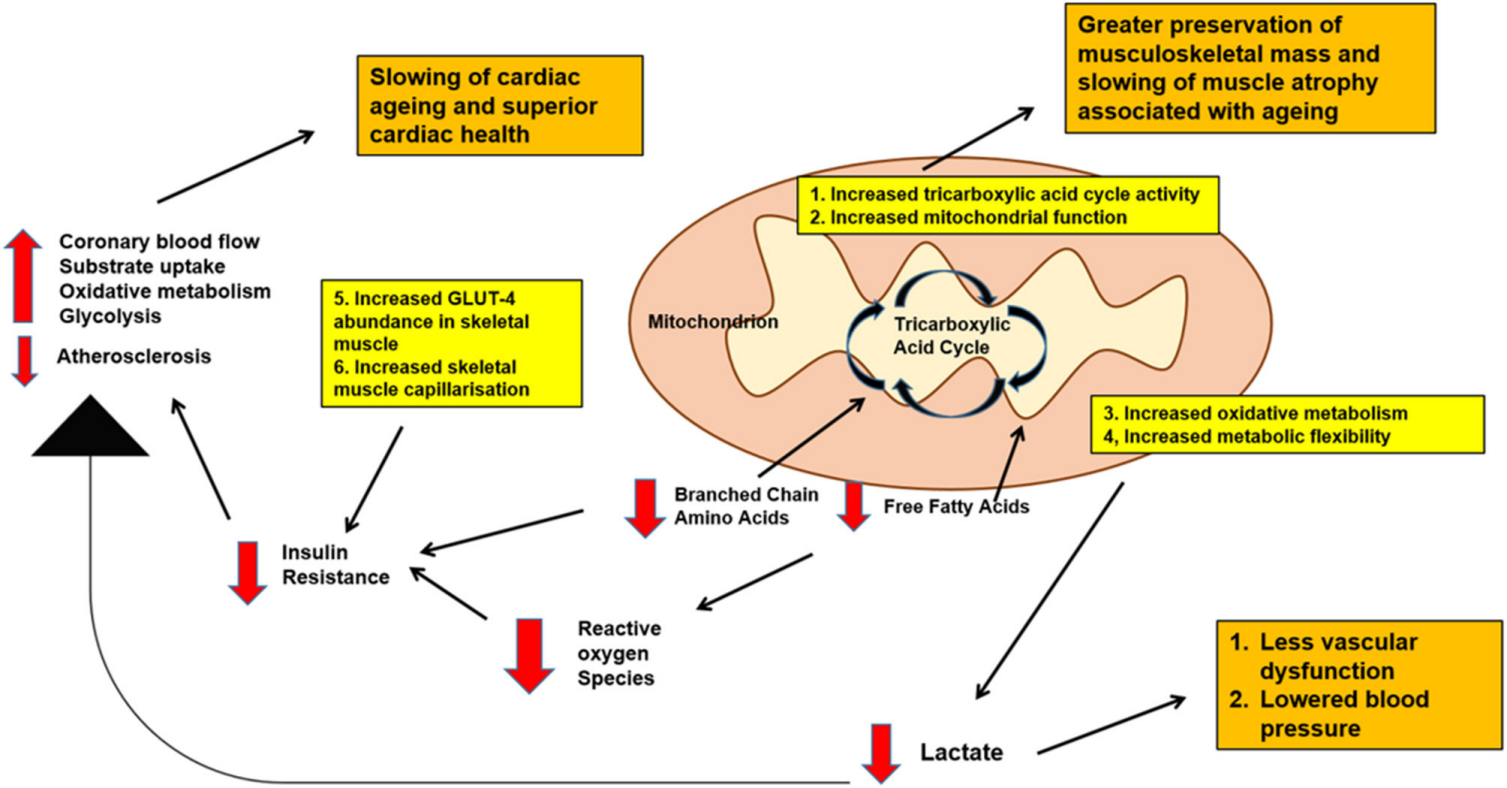
Schematic diagram of possible metabolic pathways linking physical activity, cardiovascular health and musculoskeletal function with aging. Decreased levels of branched chain amino acids and free fatty acids, decreased baseline lactate, decreased blood glucose levels as well as increased musculoskeletal mass may be expected with physical activity. All these effects are important in promoting better cardiovascular health, slowing down cardiac aging as well as maintaining good musculoskeletal mass into old age.

Figure 1 summarizes possible metabolic pathways linking physical activity, cardiovascular health and musculoskeletal function.

## Conclusions and Implications

Physical activity has clear cardiovascular and general health benefits. Human and animal model studies of heart and skeletal muscle responses to physical activity point to changes in the patterns of fuel use and mitochondrial oxidation as key components of a healthy adaptation. These changes include increased TCA cycle activity (16) and better coordination between fuel processing and TCA cycle activity (30). In contrast, heart failure and aging-related frailty are associated with accumulation of fatty acid fuel intermediates (31), mismatch between fuel supply and TCA cycle, reduced TCA cycle activity (30) and finally greater reliance on non-oxidative glucose metabolism. An emerging concept is the importance of metabolic flexibility. Healthy hearts are able to switch fuel use patterns in response to available supply and immediate energy demand (42). Reduced metabolic flexibility is associated pathologic changes and this can be reversed with exercise interventions (43). In future, dynamic testing of heart and whole-body fuel use may be an important component of assessing cardiovascular health and response to exercise interventions. We also recognize that age itself exerts a major impact on the metabolome that is separate from the effects of physical activity.

We acknowledge a limitation of our review that a cardiac-centric view of the impact of physical activity in the absence of information from other organs such as the liver, skeletal muscle and brain may limit the interpretation of the beneficial effects of physical activity at the cardiovascular and organism levels (2). In addition, our work mainly focuses on leisure time physical activity. Interestingly, it has been shown that while leisure time physical activity is associated with reduced major adverse cardiac events (MACE), occupational physical activity is instead associated with increased MACE instead. This may be due to the associated with occupational physical activity with fatigue and insufficient recovery, as well as more static and constrained activity as compared to leisure physical activity (63).

Despite the current advances in the understanding of metabolomics and its relation to cardiovascular health, there have been limited cohort studies or randomized control trials (RCTs) to provide good evidence for the role of physical activity in altering an individual's metabolomic profile, and how this change in metabolomic profile eventually affects cardiovascular health. Findings from such studies can potentially be used by clinicians to titrate exercise regimens based on individually tailored cardiovascular outcomes. These individualized exercise regimens can be prescribed even to healthy individuals, thereby reducing the cardiovascular disease burden in healthcare systems worldwide. Finally, while our review is limited to studying the effect of metabolomics in cardiovascular health, we also recognize that there has been increasing interest in studying multiple-omics techniques to gain greater insights into cardiovascular health. This would include combining genomics, transcriptomics, proteomics and metabolomics which may form a more comprehensive picture of the factors involved in cardiovascular health and heart failure as compared to either one alone (64, 65).

## Author Contributions

RL and AK conceptualized, wrote, and approved the final version of this work.

## Funding

AK received grant support from the National Medical Research Council of Singapore (MOH-000153), Hong Leong Foundation, Duke-NUS Medical School, Estate of Tan Sri Khoo Teck Puat and Singhealth Foundation.

## Conflict of Interest

The authors declare that the research was conducted in the absence of any commercial or financial relationships that could be construed as a potential conflict of interest.

## Publisher's Note

All claims expressed in this article are solely those of the authors and do not necessarily represent those of their affiliated organizations, or those of the publisher, the editors and the reviewers. Any product that may be evaluated in this article, or claim that may be made by its manufacturer, is not guaranteed or endorsed by the publisher.
